# PIPE‐cloned human IgE and IgG4 antibodies: New tools for investigating cow's milk allergy and tolerance

**DOI:** 10.1111/all.14604

**Published:** 2020-10-14

**Authors:** Christina L. Pranger, Judit Fazekas‐Singer, Verena K. Köhler, Isabella Pali‐Schöll, Alessandro Fiocchi, Sophia N. Karagiannis, Olatz Zenarruzabeitia, Francisco Borrego, Erika Jensen‐Jarolim

**Affiliations:** ^1^ The Interuniversity Messerli Research Institute of the University of Veterinary Medicine Vienna Medical University Vienna and University Vienna Vienna Austria; ^2^ Institute of Pathophysiology and Allergy Research Centre of Pathophysiology, Infectiology and Immunology Medical University of Vienna Vienna Austria; ^3^ Institute of Science and Technology Austria Klosterneuburg Austria; ^4^ Allergy Division Pediatric Hospital Bambino Gesù IRCCS Roma Italy; ^5^ St. John's Institute of Dermatology School of Basic & Medical Biosciences King's College London Guy's Hospital London UK; ^6^ Breast Cancer Now Research Unit School of Cancer & Pharmaceutical Sciences King's College London London UK; ^7^ Immunopathology Group Biocruces Bizkaia Health Research Institute Barakaldo Spain; ^8^ Ikerbasque Basque Foundation for Science Bilbao Spain

**Abbreviations**
AIT: allergen immunotherapy
BAT: basophil activation test
BLG: beta‐lactoglobulin
CM: cow’s milk
CMA: cow’s milk allergy
D1: name of engineered antibodies due to their published, variable regions
ELISA: enzyme‐linked immunosorbent assay
FA‐AIT: food allergen immunotherapy
IL: interleukin
ISAC: Immuno‐Solid phase Allergy Chip
OIT: oral immunotherapy
PBMC: peripheral blood mononuclear cells
PIPE cloning: polymerase incomplete primer extension cloning
RT: room temperature
SDS‐PAGE: sodium dodecyl sulphate‐polyacrylamide gel electrophoresis
TBS: Tris‐buffered saline
TBS‐T: TBS‐Tween
UHT milk: ultra‐high temperature processed milk

To the Editor,

Cow's milk (CM) allergy (CMA) is defined as an immune‐mediated adverse response to CM proteins.

2% to 3% of children are suffering from CMA, but many develop natural tolerance after 3‐4 years.^1^ ^,S01^ Food allergen immunotherapy (FA‐AIT) applying increasing antigen doses (oral immunotherapy, OIT) can contribute to improvement of CMA,^S02^ with variable clinical efficacy,^2^ ^,S03^ but immunologically often resulting in decreased specific IgE levels and increased specific IgG4 levels^S04,S05^. IgG4 is (a) anti‐inflammatory as it does not activate the complement system; (b) bi‐specific due to fab‐arm exchange and, thus, has less crosslinking capacity than IgE, but has (c) blocking capacity.^3^ The interplay of IgE and IgG4 may hence be decisive for the immune balance in CMA.

Antibodies of different isotypes with the same variable region could be essential in molecular studies of the mechanism underlying CMA and might shed light onto the function of specific IgG4 antibodies during the acquisition of tolerance.

Polymerase Incomplete Prime Extension (PIPE) cloning is a cutting‐edge technique to engineer antibodies against various targets and facilitates simple exchange of the constant regions to increase the range of subtypes of antibodies.^4^, ^5^, ^6^ In this study, we combined variable regions from the high‐affinity IgE antibody “D1”^7^ (KD = 1.4 × 10^−9^ M; *k*_on_ = 1.4 × 10^6^, *k*_off_ = 1.54 × 10^−3^)^S06^ specific for the major CM allergen beta‐lactoglobulin (BLG), with the constant domains of IgE or IgG4 antibodies (Figure 1A). Correct antibody assembly was confirmed by SDS‐PAGE in comparison with commercial antibodies of the same isotype (Figure 1B). The exclusive specificity of D1 antibodies was proven in ELISA (Figure 1C), in ISAC112 microarray (Figure 1D,E) and in ImmunoCAP (data not shown). Antibodies were furthermore species‐specific to bovine BLG when testing milk samples from various animal species and showed a sensitivity comparable to a commercial anti‐BLG‐ELISA (Figure S1).

**FIGURE 1:**
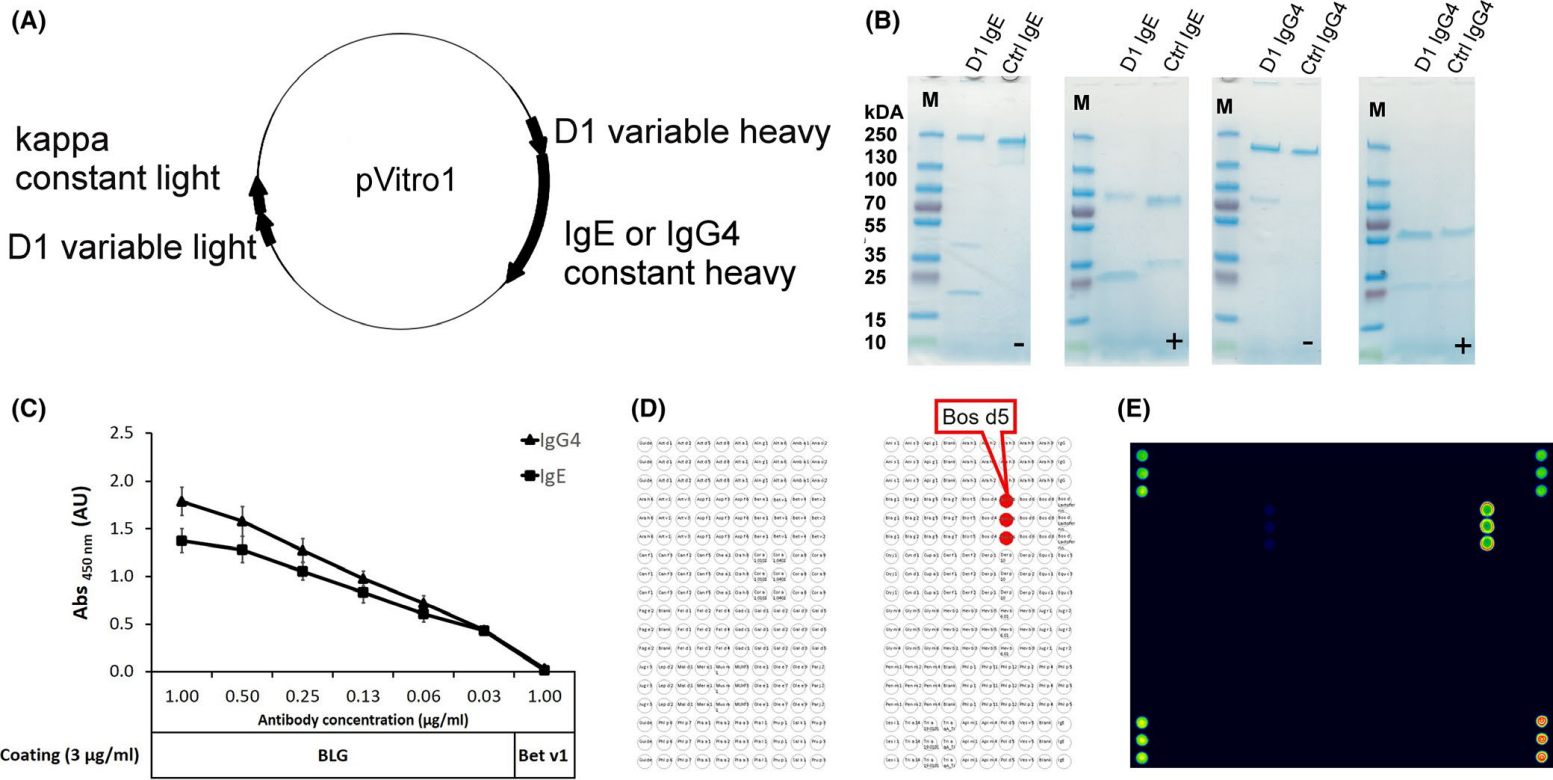
Correct assembly and BLG‐specific binding of PIPE‐cloned D1 IgE and IgG4 antibodies. A, Schematic overview of the pVitro1 antibody constructs for the production of D1 IgE and IgG4. B, SDS‐PAGE of D1 antibodies in comparison with commercial isotype antibodies under nonreducing (−) and reducing condition (+). C, Specificity of D1 anti‐BLG antibodies in an ELISA. 3 μg/mL of BLG or Bet v 1 (control) were coated onto ELISA plates, and binding of respective D1 antibodies at different concentrations was determined. Data represent the mean of three independent experiments, and error bars indicate SD. D, Overview of ISAC112 microarray layout. E, Specific binding of D1 IgE to BLG (Bos d 5) in ISAC112

We next applied our antibodies for the quantification of BLG‐specific IgE and IgG4 antibodies in serum of milk allergic patients (n = 9) and sensitised but tolerant patients (n = 8). Allergic children had significantly higher levels of IgE (*P* < .01), but no differences in the IgG4 levels were seen between the groups (Figure S2), in accordance with previous studies.^8^ Our data suggest that the PIPE‐cloned antibodies may be useful for a precise quantification of allergen‐specific IgE and IgG4. This is advantageous, as specific IgE is usually quantified by comparing the reactivity to IgE absorbed to a solid phase, likely leading to an underestimation of specific IgE and semi‐quantitative results.

Basophil activation test (BAT) is a reliable method to evaluate functional capacities of antibodies, allowing at least an approximation to the real‐life situation of an allergic patient. When IgE‐stripped basophils from healthy donors were sensitised with D1 IgE antibodies and triggered with BLG, basophil activation levels comparable to primary basophils from milk allergic patients in other studies were achieved.^9^ Activation of the basophils, measured by the surface expression of the activation marker CD63, was specifically seen after stimulation with BLG, but not with the control milk allergen casein (*P* < .05; Figure 2A) and this was associated with increased production of the intracellular Th_2_ cytokines IL‐4 (*P* = .0187) and IL‐13 (*P* = .0048; Figure 2B). Overall, the results propose that our IgE antibodies are functional and may be useful tools to standardise BAT assays to achieve comparability between different laboratories.

**FIGURE 2:**
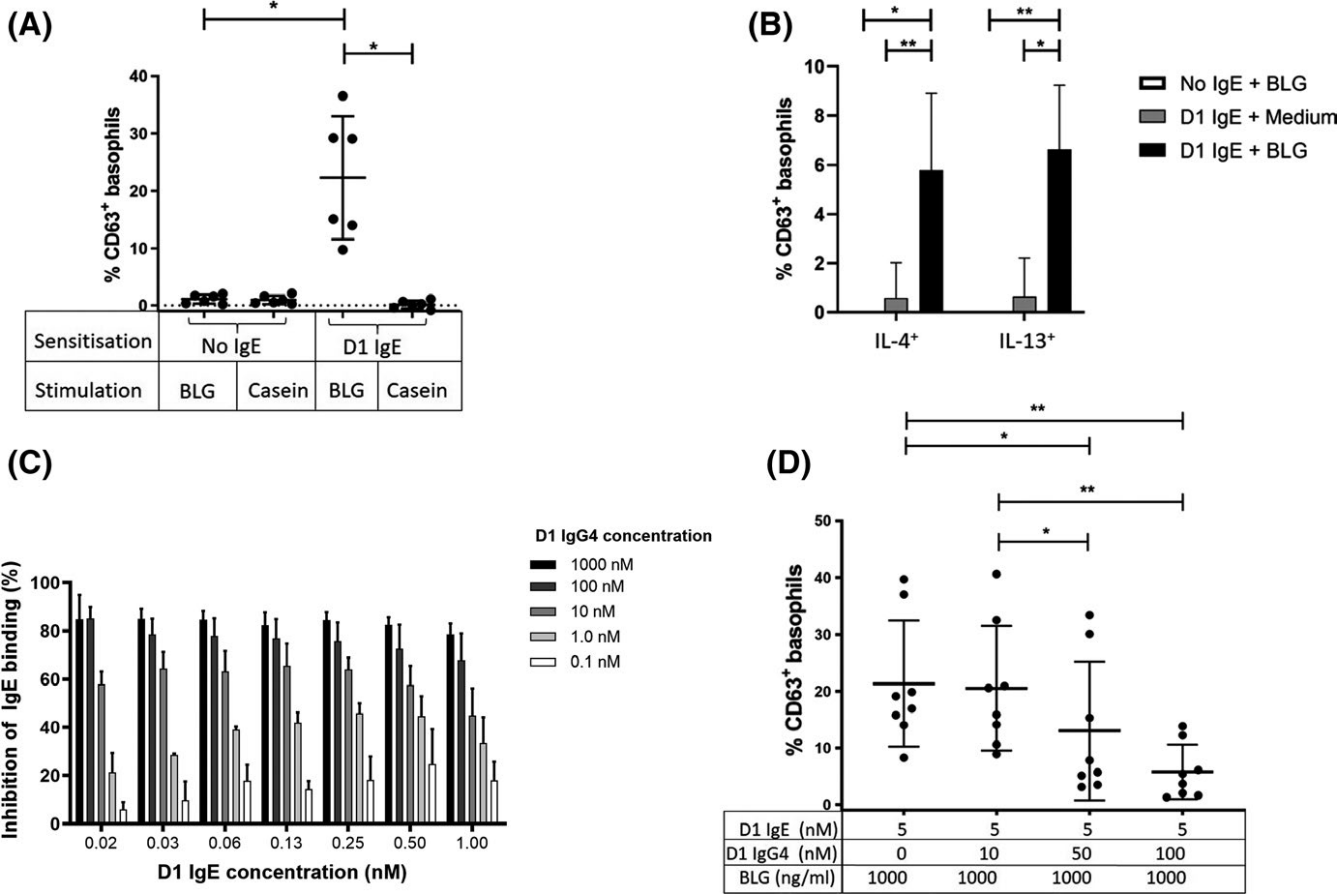
Functionality testing of D1 IgE in passively sensitised basophils: CD63 upregulation and intracellular cytokine production and blocking capacity testing of D1 IgG4: blocking of IgE binding in ELISA and inhibition of IgE crosslinking in BAT in a concentration‐dependent manner. A, Stripped and IL‐3 primed basophils from six healthy donors, native or passively sensitized with 5 nmol/L D1 IgE were triggered with BLG, or casein control, both at 1000 ng/mL. Control experiments were performed in the absence of IgE. Normal distribution was confirmed by Shapiro‐Wilk normality test and differences between the groups assessed with repeated measures ANOVA with Greenhouse‐Geisser correction and Sidak multiple comparison test: *F*(1.022, 5.110) = 24.01, *P* = .0041. Gating strategy: See Figure S3, supplement. B, IgE‐stripped and IL‐3 primed basophils of healthy donors, native or passively sensitized with 5 nmol/L D1 IgE, were stimulated with 1000 ng/mL BLG or medium control. *Y*‐axis: percentage of CD63^+^ cells, *x*‐axis: levels of intracellularly expressed cytokines. Differences in the groups were statistically analysed using two‐way ANOVA with Greenhouse‐Geisser correction: *F*(1.276, 6.382) = 45.27, *P* = .0003 and a Bonferroni multiple comparison test. *P < .05, **P < .01, and ***P < .001. Gating strategy: See Figure S3, supplement. C, BLG was coated onto ELISA plates, D1 IgG4 added in increasing concentrations (see colour code for white to black columns), and then D1 IgE added in various concentration as shown on *x*‐axis. Bound IgE was detected by HRP‐labelled anti‐human‐IgE antibody. Data represents the mean ± SD of three independent experiments. D, Percentage of CD63^+^ activated basophils in eight healthy donors (*y*‐axis) after IgE‐stripping, IL‐3 priming, sensitising with 5 nmol/L BLG‐specific D1 IgE and subsequent stimulation with 1000 ng/mL BLG alone, or BLG mixed with 0‐100 nmol/L D1 IgG4 antibodies, as described in the *x*‐axis. Significant differences were assessed with the Friedmann test: χ^2^(4) = 19.80, *P* = .0002, n = 8 and Dunn's multiple comparison test *P < .05, **P < .01, and ***P < .001. Gating strategy: see Figure S3, supplement

The blocking capacity of D1 IgG4 antibodies was investigated in an ELISA. As expected, the D1 IgG4, harbouring the same variable region, could decrease D1 IgE binding to plate‐bound BLG in a concentration‐dependent manner (Figure 2C). In accordance with these results, D1 IgG4 inhibited the activation of IgE‐stripped, IL‐3 primed, D1 IgE‐sensitised and BLG‐stimulated basophils from healthy donors in a dose‐dependent manner. The addition of 50 nmol/L (*P* = .0402) or 100 nmol/L D1 IgG4 (*P* = .0029) significantly decreased the percentage of CD63^+^ basophils (Figure 2D). In agreement with others, our findings confirmed the importance of the IgG4:IgE‐ratio, as no inhibition was achieved with the lowest concentration of D1 IgG4 (10 nmol/L). In line with our D1 IgE/D1 IgG4‐model, overlapping epitope‐binding of IgG4 and IgE antibodies may play an essential role in acquiring tolerance.^8^ At the same time, this is also the limitation of our molecular model: the situation in allergic patients with polyclonal antibodies, binding to various epitopes on the allergen, is more complex. However, in a parallel study, with a PIPE‐cloned monoclonal IgG4 against the major birch pollen allergen Bet v 1, we were able to demonstrate its blocking capacity using the sera from birch allergic patients.^S07^

Whether the IgG4 antibodies could be applied as passive immunotherapy in cow milk allergic patients depends on a thorough risk‐benefit analysis, but studies in cat allergy promise that this might be possible in the future.^S08^

Our antibodies have so far proven valuable not only for the quantification of BLG and BLG‐specific human serum antibodies, but also for functional studies. With these tools, some questions could be addressed in the future: Are IgG4 antibodies involved in the establishment of *persisting* tolerance, or in primary tolerance to milk? Contrasting results about the efficacy of oral immunotherapies for CMA may be explained by differences in the methods regarding the duration of the intervention, dosages of the allergen or the composition of the milk. The PIPE‐cloned antibodies presented here may add to the comparability of such studies between laboratories. Another application of the D1 antibodies may be in studies on the effect of milk processing on immune responses. Food processing can massively influence the structure of the allergens and therefore alter their allergenicity.^S09,S10^ Especially during heat processing, important BLG epitopes are lost, but IgE binding is significantly increased after pasteurisation.^S11^ Overall, the generated antibodies may shed light on milk allergenicity, bring critical insight into the development of CMA and help developing prevention strategies.

## CONFLICT OF INTEREST

EJJ is founder and shareholder of Biomedical Int. R+D GmbH, Vienna, Austria, and business partner of Bencard Allergie GmbH, Germany as well as AllergyTherapeutics Ltd., UK, but has no COI in relation to the presented work. EJJ also holds patents in lipocalins including BLG for allergen immunotherapy. SNK is founder and shareholder of Epsilogen Ltd. and holds patents on IgE antibodies for cancer therapy. AF is conducting research supported by Danone SA and Hipp GmbH. He has been in the advisory boards of Abbott SA and Hipp GmbH over the last two years. The other authors have no potential conflicts of interest to disclose.

## Supporting information

Figure S1

Figure S2

Figure S3

Supplementary Material
