# Supplementary figures and images for: PIPE‐cloned human IgE and IgG4 antibodies: New tools for investigating cow's milk allergy and tolerance
Source: Allergy. 2020 Oct 14;76(5):1553–6. doi: 10.1111/all.14604 (PMC8247298; doi:10.1111/all.14604)

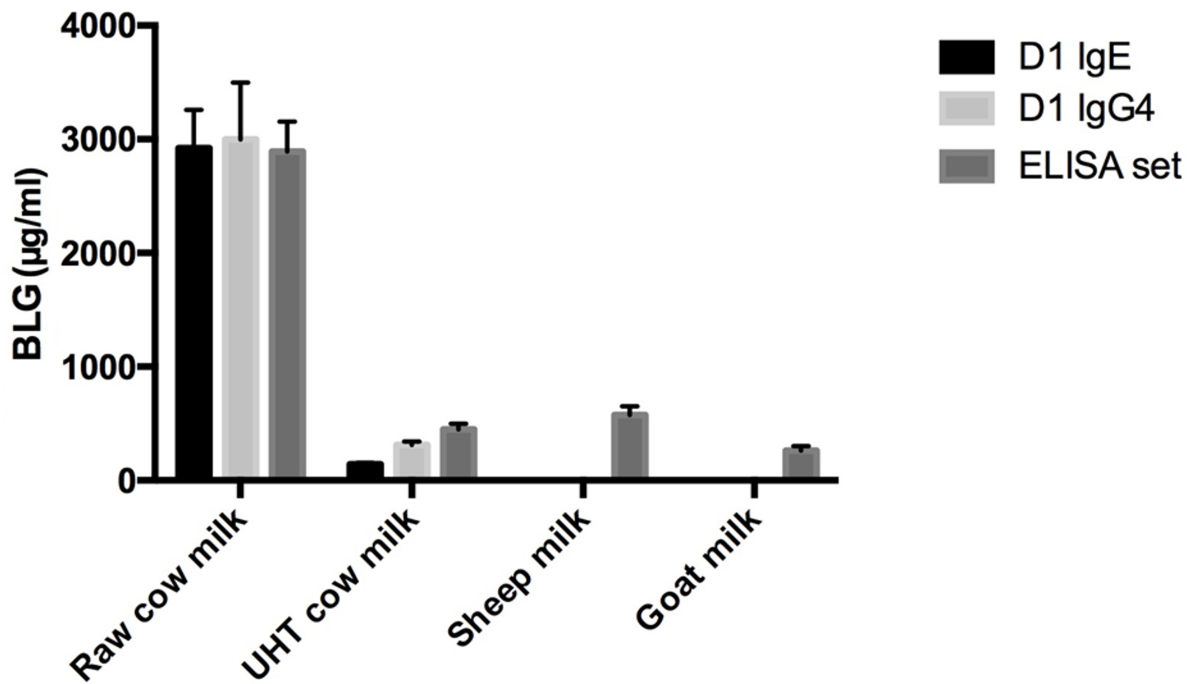

Supplement: Supplementary file 1 — Figure S1 [file ALL-76-1553-s004.pdf]

**A**

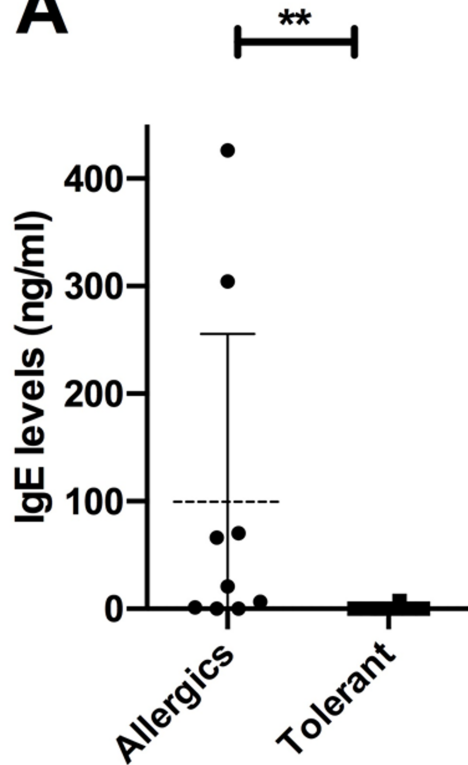

# B

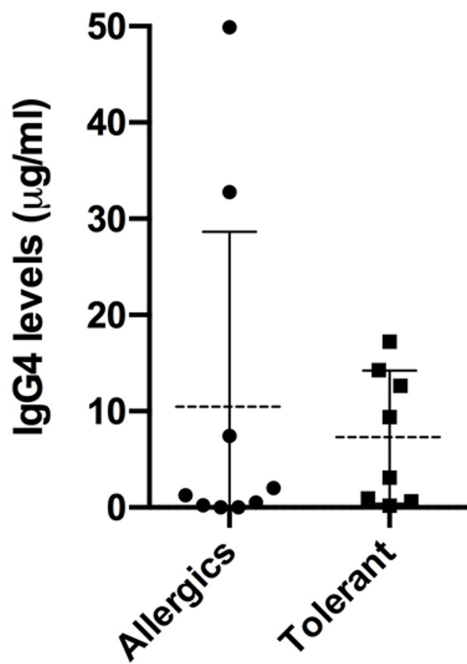

Supplement: Supplementary file 2 — Figure S2 [file ALL-76-1553-s003.pdf]

# Main population

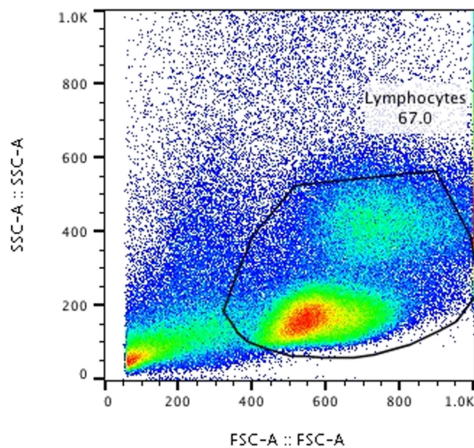

# single cells

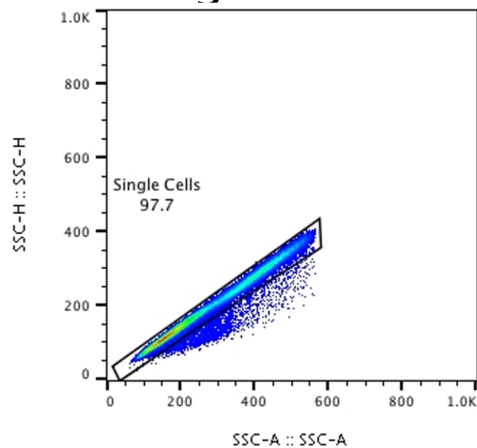

# live cells

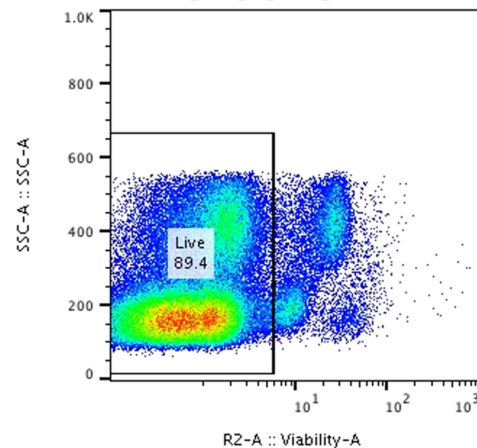

# Basophils CD123+FcεRI+

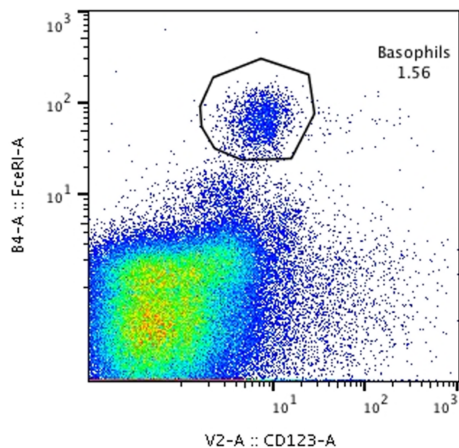

# Basophils HLA-DR-

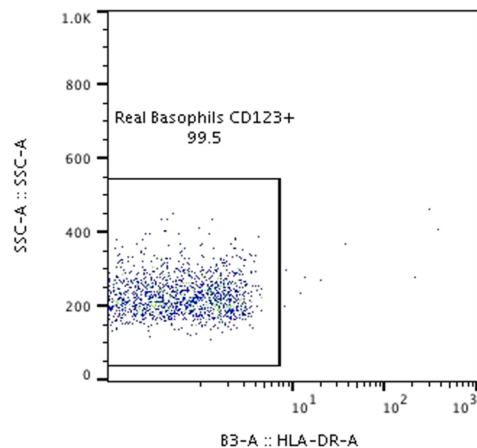

# Basophils CD63+

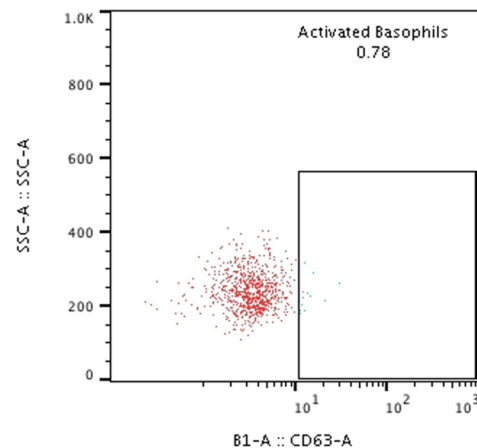

Supplement: Supplementary file 3 — Figure S3 [file ALL-76-1553-s001.pdf]
