# Supplementary material for: PIPE‐cloned human IgE and IgG4 antibodies: New tools for investigating cow's milk allergy and tolerance
Source: Allergy. 2020 Oct 14;76(5):1553–6. doi: 10.1111/all.14604 (PMC8247298; doi:10.1111/all.14604)
Supplement: Supplementary file 4 — Supplementary Material [file ALL-76-1553-s002.docx]

**Supplementary Material**

**Cell cultivation and maintenance**

Expi293F cells (#A14527, Thermo Fisher Scientific) were cultivated in 30 ml Expi293 Expression Medium (#A1435101, Gibco, Thermo Fisher Scientific) at 37 °C, 8 % CO_2_, according to the manufacturer’s instructions. PBMCs of healthy patients were incubated in complete Iscove’s Modified Dulbecco Medium (#12440-053, Gibco, Thermo Fisher Scientific); supplemented with 1 % GlutaMAX™ (#35050-038, Gibco, Thermo Fisher Scientific) and 10 % AB serum (# 20726, Male AB Serum, Innovative Research, Novi, MI, USA).

**PIPE cloning and antibody production**

Published variable region sequences (Table S1) of an IgE antibody specific for BLG^7^ were combined with the human ε and γ4 constant regions to create different subtypes of antibodies binding to the same allergen epitope. pUC57 plasmids with an ampicillin resistance gene carrying the heavy and light chain of the variable region were purchased from BioCat GmbH (Heidelberg, Germany); vectors with the heavy and light chains of the constant regions for IgE and IgG4 (pVitro1, hygromycin resistance) were provided by King’s college, London, UK. Variable and constant regions were amplified in four separate PIPE PCRs as described previously^4,5^.

Briefly, 1 µl template DNA (10 ng/µl) was mixed with 2.5 µl of suitable forward and reverse primer (10 µM), 25 µl 2x Phusion Flash High-Fidelity PCR master mix (# F548S, Thermo Fisher Scientific) and 19 µl nuclease-free water. The reaction was started as initial denaturation at 98 °C for 30 s and then 30 cycles of denaturation at 98 °C for 10 s, annealing at 60 °C for 15 s, extension at 72 °C for the time indicated in table S2, followed by a cool down to 4 °C.

**Table S1.** Light chain and heavy chain sequences of D1 IgE and IgG4

| Light chain sequence:  *Italic: human leader,*  **bold: D1 V_L_ sequence,**  underlined:  hu κ equence,  ***bold italic: stop codon*** | *ATGTTGCCATCACAACTCATTGGGTTTCTGCTGCTCTGGGTTCCAGCTAGCCGCGGT***GACATCGTGATGACACAGAGCCCTAGCAGCCTGTCTGCCAGCGTGGGAGACAGAGTGACCATCACCTGTAGAGCCAGCCAGGGCATCAGCTCTAGACTGGCCTGGTATCAGCAGAAGCCTGGCAAGGCCCCTAAGCTGCTGATCTATGCCGCTAGCTCTCTGCAGTCTGGCGTGCCCTCTAGATTTTCTGGCAGCGGCTCTGGCACCGAGTTCACCCTGACCATATCTAGCCTGCAGCCTGAGGACTTCGCCACCTACTACTGCCAGCAGTACCACAGCTACCCCTGGACATTTGGCCAGGGCACCAAGGTGGAAATC**AAGCGTACGGTGGCGGCGCCATCTGTCTTCATCTTCCCGCCATCTGATGAGCAGTTGAAATCTGGAACTGCCTCTGTTGTGTGCCTGCTGAATAACTTCTATCCCAGAGAGGCCAAAGTACAGTGGAAGGTGGATAACGCCCTCCAATCGGGTAACTCCCAGGAGAGTGTCACAGAGCAGGACAGCAAGGACAGCACCTACAGCCTCAGCAGCACCCTGACGCTGAGCAAAGCAGACTACGAGAAACACAAAGTCTACGCCTGCGAAGTCACCCATCAGGGCCTGAGCTCGCCCGTCACAAAGAGCTTCAACAGGGGAGAGTGT***TGA*** |
| --- | --- |
| IgE heavy chain sequence:  *Italic: human leader,*  **bold: D1 V_H_ sequence,**  underlined:  hu ε equence,  ***bold italic: stop codon*** | *ATGGACTGGACCTGGAGGATCCTCTTCTTGGTGGCGGCCGCCACAGGCGCGCACTCC***CAGGTGTCCCTGAGAGAATCTGGCGGAGGACTGGTGCAGCCTGGCAGATCTCTGAGACTGAGCTGTACCGCCAGCGGCTTCACCTTTAGACACCACGGCATGACCTGGGTCCGACAGGCTCCTGGAAAAGGCCTGGAATGGGTCGCCTCTCTGTCTGGCTCTGGCACCAAGACACACTTCGCCGACTCTGTGAAGGGCAGATTCACCATCAGCCGGGACAACAGCAACAACACCCTGTACCTGCAGATGGACAACGTGCGCGACGAGGACACCGCCATCTACTATTGTGCCAAGGCCAAGAGAGTGGGCGCCACCGGCTATTTCGATCTGTGGGGAAGAGGCACCCTGGTCACCGTTTCTTCT**GCTAGCACACAGAGCCCATCCGTCTTCCCCTTGACCCGCTGCTGCAAAAACATTCCCTCCAATGCCACCTCCGTGACTCTGGGCTGCCTGGCCACGGGCTACTTCCCGGAGCCGGTGATGGTGACCTGGGACACAGGCTCCCTCAACGGGACAACTATGACCTTACCAGCCACCACCCTCACGCTCTCTGGTCACTATGCCACCATCAGCTTGCTGACCGTCTCGGGTGCGTGGGCCAAGCAGATGTTCACCTGCCGTGTGGCACACACTCCATCGTCCACAGACTGGGTCGACAACAAAACCTTCAGCGTCTGCTCCAGGGACTTCACCCCGCCCACCGTGAAGATCTTACAGTCGTCCTGCGACGGCGGCGGGCACTTCCCCCCGACCATCCAGCTCCTGTGCCTCGTCTCTGGGTACACCCCAGGGACTATCAACATCACCTGGCTGGAGGACGGGCAGGTCATGGACGTGGACTTGTCCACCGCCTCTACCACGCAGGAGGGTGAGCTGGCCTCCACACAAAGCGAGCTCACCCTCAGCCAGAAGCACTGGCTGTCAGACCGCACCTACACCTGCCAGGTCACCTATCAAGGTCACACCTTTGAGGACAGCACCAAGAAGTGTGCAGATTCCAACCCGAGAGGGGTGAGCGCCTACCTAAGCCGGCCCAGCCCGTTCGACCTGTTCATCCGCAAGTCGCCCACGATCACCTGTCTGGTGGTGGACCTGGCACCCAGCAAGGGGACCGTGAACCTGACCTGGTCCCGGGCCAGTGGGAAGCCTGTGAACCACTCCACCAGAAAGGAGGAGAAGCAGCGCAATGGCACGTTAACCGTCACGTCCACCCTGCCGGTGGGCACCCGAGACTGGATCGAGGGGGAGACCTACCAGTGCAGGGTGACCCACCCCCACCTGCCCAGGGCCCTCATGCGGTCCACGACCAAGACCAGCGGCCCGCGTGCTGCCCCGGAAGTCTATGCGTTTGCGACGCCGGAGTGGCCGGGGAGCCGGGACAAGCGCACCCTCGCCTGCCTGATCCAGAACTTCATGCCTGAGGACATCTCGGTGCAGTGGCTGCACAACGAGGTGCAGCTCCCGGACGCCCGGCACAGCACGACGCAGCCCCGCAAGACCAAGGGCTCCGGCTTCTTCGTCTTCAGCCGCCTGGAGGTGACCAGGGCCGAATGGGAGCAGAAAGATGAGTTCATCTGCCGTGCAGTCCATGAGGCAGCGAGCCCCTCACAGACCGTCCAGCGAGCGGTGTCTGTAAATCCCGGTAAA***TGA*** |
| IgG_4_ heavy chain sequence  *Italic: human leader,*  **bold: D1 V_H_ sequence,**  underlined:  hu γ_4_ sequence,  ***bold italic: stop codon*** | *ATGGACTGGACCTGGAGGATCCTCTTCTTGGTGGCGGCCGCCACAGGCGCGCACTCC***CAGGTGTCCCTGAGAGAATCTGGCGGAGGACTGGTGCAGCCTGGCAGATCTCTGAGACTGAGCTGTACCGCCAGCGGCTTCACCTTTAGACACCACGGCATGACCTGGGTCCGACAGGCTCCTGGAAAAGGCCTGGAATGGGTCGCCTCTCTGTCTGGCTCTGGCACCAAGACACACTTCGCCGACTCTGTGAAGGGCAGATTCACCATCAGCCGGGACAACAGCAACAACACCCTGTACCTGCAGATGGACAACGTGCGCGACGAGGACACCGCCATCTACTATTGTGCCAAGGCCAAGAGAGTGGGCGCCACCGGCTATTTCGATCTGTGGGGAAGAGGCACCCTGGTCACCGTTTCTTCT**GCTAGCACCAAGGGCCCATCCGTCTTCCCCCTGGCGCCCTGCTCCAGGAGCACCTCCGAGAGCACAGCCGCCCTGGGCTGCCTGGTCAAGGACTACTTCCCCGAACCGGTGACGGTGTCGTGGAACTCAGGCGCCCTGACCAGCGGCGTGCACACCTTCCCGGCTGTCCTACAGTCCTCAGGACTCTACTCCCTCAGCAGCGTGGTGACCGTGCCCTCCAGCAGCTTGGGCACGAAGACCTACACCTGCAACGTAGATCACAAGCCCAGCAACACCAAGGTGGACAAGAGAGTTGAGTCCAAATATGGTCCCCCATGCCCATCATGCCCAGCACCTGAGTTCCTGGGGGGACCATCAGTCTTCCTGTTCCCCCCAAAACCCAAGGACACTCTCATGATCTCCCGGACCCCTGAGGTCACGTGCGTGGTGGTGGACGTGAGCCAGGAAGACCCCGAGGTCCAGTTCAACTGGTACGTGGATGGCGTGGAGGTGCATAATGCCAAGACAAAGCCGCGGGAGGAGCAGTTCAACAGCACGTACCGTGTGGTCAGCGTCCTCACCGTCCTGCACCAGGACTGGCTGAACGGCAAGGAGTACAAGTGCAAGGTCTCCAACAAAGGCCTCCCGTCCTCCATCGAGAAAACCATCTCCAAAGCCAAAGGGCAGCCCCGAGAGCCACAGGTGTACACCCTGCCCCCATCCCAGGAGGAGATGACCAAGAACCAGGTCAGCCTGACCTGCCTGGTCAAAGGCTTCTACCCCAGCGACATCGCCGTGGAGTGGGAGAGCAATGGGCAGCCGGAGAACAACTACAAGACCACGCCTCCCGTGCTGGACTCCGACGGCTCCTTCTTCCTCTACAGCAGGCTAACCGTGGACAAGAGCAGGTGGCAGGAGGGGAATGTCTTCTCATGCTCCGTGATGCATGAGGCTCTGCACAACCACTACACACAGAAGAGCCTCTCCCTGTCTCTGGGTAAA***TGA*** |

**Table S2.** PIPE PCR details and primers for amplification of the four fragments

| Name | Template | Fwd primer | Rev primer | Fragment  size (bp) | Extension time (s) |
| --- | --- | --- | --- | --- | --- |
| Frag. 1 – IgE | D1VH | GCCACAGGCGCGCACTCCCAGGTGTCCCTGAGAGAAT | ATGGGCTCTGTGTGCTAGCAGAAGAAACGGTGACCAGGGT | 360 | 5 |
| Frag. 2 – IgE | E | GCTAGCACACAGAGCCCATCCGTCTTCCCCTTGACCCGCTGCTGCA | ACCGCGGCTAGCTGGAACCCAGAGCAGCAGAAACCCAATGAGTTG | 4076 | 56.6 |
| Frag. 1 – IgG4 | D1VH | GCCACAGGCGCGCACTCCCAGGTGTCCCTGAGAGAAT | AAGACGGATGGGCCCTTGGTGCTAGCAGAAGAAACGGTGACCAG | 376 | 5 |
| Frag. 2 – IgG4 | G4 | GCTAGCACCAAGGGCCCATCCGTCTTCCCCCTGGC | ACCGCGGCTAGCTGGAACCCAGAGCAGCAGAAACCCAATGAGTTG | 3773 | 52.4 |
| Frag. 3 – all | D1VL | TTCCAGCTAGCCGCGGTGACATCGTGATGACACAGAGCCCTAGC | CGCCGCCACCGTACGCTTGATTTCCACCTTGGTGCCCTGGCCAA | 313 | 4.3 |
| Frag. 4 – all | Κ for  E/G4 | CGTACGGTGGCGGCGCCATCTGTCTTCATCTTCCCGCCATCTG | GGAGTGCGCGCCTGTGGCGGCCGCCACCAAGAAGAGGATC | 4126 | 57.3 |

PCR products were digested with DpnI (#R0176S, New England Biolabs (NEB), Ipswich, MA, USA) and analysed on a 1 % agarose gel. Fragments were ligated by mixing 100 ng/µl of the four PCR products in a 1:1:1:1 ratio and incubated for 1 h at RT. Transformation into competent *E. coli* top 10 (#C3019H, NEB) cells was done according to the manufacturer’s instructions and bacterial colonies were cultivated on agar plates supplemented with 200 µg/ml hygromycin B (#1287, Carl Roth, Karlsruhe, Germany) overnight at 37 °C. Colony PCR was conducted to check for heavy and light chain inserts. DNA was released from bacterial colonies via heat treatment (95 °C for 10 min) and mixed with 0.5 µl suitable forward and reverse primers, 5 µl 2x Phusion Flash High-Fidelity PCR master mix (#F548S, Thermo Fisher Scientific). Colony PCR program was started with an initial denaturation at 98 °C for 30s, followed by 30 cycles of denaturation at 98 °C for 10 s, annealing at 60 °C or 15s, extension at 72 °C for 7 s and ended in a final extension step at 72 °C for 10 min and a cool down to 4 °C. Sequences were confirmed via cycle sequencing (Mix2seq overnight kit; Eurofins Genomics, Ebersberg, Germany). Finally, Expi293F cells (#A14527, Thermo Fisher Scientific, Waltham, MA, USA) were transfected with the plasmids and 50 µg/ml hygromycin B (#1287, Carl Roth) was added 72 h after transfection to maximise antibody yield. After seven days of culture, antibodies were purified by affinity chromatography using either a HiTrap KappaSelect column (#17545811, GE healthcare, Chicago, IL, USA) for IgE, or a HiTrap Protein A column (#17040201, GE healthcare) for IgG4 antibodies. Antibodies were eluted with either 0.1 M glycine (pH 2.5, IgE) or 0.1 M citric acid (pH 3, IgG4) and pH was immediately neutralised by adding 100 µl of Tris-HCl (pH 9.3). Antibody concentration was measured by UV absorption at 280 nm with a UV/VIS spectrophotometer (DeNovix DS-11 FX+, DeNovix Inc.), using the extinction coefficients from the protein sequence.

**SDS-PAGE**

2 µg of the PIPE-cloned D1 antibodies as well as isotype controls, human plasma IgE (#16-16-090705, Athens Research & Technology, Athens, GA, USA) and human myeloma IgG4 (#16-16-090707-4M, Athens Research & Technology) were diluted in PBS, mixed with 10 µl non-reducing or reducing Laemmli sample buffer, and heated for 5 min at 95 °C. Samples and markers (#26619, Page Ruler, Thermo Fischer Scientific) were loaded into Mini PROTEAN TGX gels (#4561084, Bio-Rad, Hercules, CA, USA) and electrophoretically separated for 20 min at 250 V. Gels were stained with SimplyBlue™ SafeStain (#LC6060, Thermo Fisher Scientific) according to the manufacturer’s instructions.

**Specificity ELISA**

For specificity determination, ELISA plates (Maxisorp 96-well plates, Thermo Fisher Scientific) were coated with 3 μg/ml of BLG (#L01030, Sigma‐Aldrich, St. Louis, MO, USA) or 3 μg/ml of recombinant control allergen Bet v 1 (produced as previously described^S12,S13^) in coating buffer, pH 9.6 (#421701, Biolegend, San Diego, CA, USA), overnight. Plates were washed once with 0.05 % TBS-T and blocked with 1% BSA in 0.05% TBS-T for 2 h at RT. After two washing steps, D1 IgE or D1 IgG4 were added at concentrations from 1 to 0.03 μg/ml, and incubated for one hour, at RT. After washing, HRP-labelled anti-human IgE antibodies 1:6,000 (#A18793, Invitrogen, Carlsbad, CA, USA), or anti-human IgG4 1:12,000 (#9200-05, Southern Biotech, Birmingham, AL, USA), were added and incubated for one hour at RT. After washing the reaction was developed by TMB (#00-4201-56, Invitrogen) and stopped with 1 M H_2_SO_4_. The absorbance was measured at 450 nm with an Infinite 200M PRO plate reader (Tecan, Männedorf, Switzerland).

**Sandwich ELISA for soluble BLG in milk**

1 μg/ml of a commercial anti-BLG antibody (#Ab112893, Abcam, Cambridge, UK) was coated onto ELISA plates. After washing and blocking as described above, 2.5 - 0.05 μg/ml of BLG (#L01030, Sigma‐Aldrich) as standard, raw cow milk (diluted 1:5,000 – 1:40,000), ultra-high temperature processed (UHT) cow milk, sheep milk and goat milk from a local store (1:500 – 1:1,000) were added and incubated for one hour, at RT. After washing, 1 μg/ml of D1 IgE or 0.25 μg/ml D1 IgG4 antibodies were added for one hour, RT and detected by HRP-labelled anti-human IgE and IgG4 as described above. For comparison, milk samples were tested with anti-BLG ELISA set (#E10-125, Bethyl Laboratories, Inc., Montgomery, Texas, US) according to the manufacturer´s instructions.

## **ImmunoCAP ISAC112**

For testing D1 IgE specificity, ImmunoCAP ISAC112 microarray (Thermo Fisher Scientific, WO) was used, following the manufacturer’s instructions by diluting 1 µg of the antibody in control serum obtained from a non-allergic person and devoid of specific antibodies to milk.

**Patients´ characteristics**

Blood samples of CM-allergic patients (n=3) and healthy controls (n=2) were collected in the Cruces University Hospital and provided by the Basque Biobank, Spain (http://www.biobancovasco.org). These patients presented IgE-mediated allergy symptoms, positive skin prick test responses to milk, and with specific IgE to cow’s milk in ImmunoCAP (Table S3).

Paediatric patients diagnosed with CMA by the Alergologia Dept., Ospedale Pediatrico Bambino Gesù, Rome, Italy were all sensitized to cow´s milk (n=14), with one group (n=6) reacting positive to an oral milk challenge and the other sensitised but tolerant group not reacting to oral milk challenge (n=8) (see patients´ characterisation in Table 3). The serum were retrospectively collected, after the informed consent from the patients were signed, in accordance with the Helsinki Declaration of 1975. Experiments were performed under approval of the ethical committee of the Pediatric Hospital Bambino Gesù IRCCS, Rome and by the Ethical Committee of Clinical Research of the Basque Country (CEIC-E) (PI2015182 and PI2016156).

**Table S3: Patients’ characteristics**

| **Patient** | **Status** | **Age**  **(months)** | **Sex** | **IgE to milk (ImmunoCAP, kU/L)** |
| --- | --- | --- | --- | --- |
| 1 | healthy control | 108 | F | nA |
| 2 | healthy control | 48 | F | nA |
| 3 | allergic ^a)^ | 72 | F | 46.3 |
| 4 | allergic | 16 | F | > 100.0 |
| 5 | allergic | 84 | M | 62.5 |
| 6 | allergic | 123 | M | 2.6 |
| 7 | allergic | 22 | F | 4.8 |
| 8 | allergic | 40 | M | 1.1 |
| 9 | allergic | 84 | M | 53.8 |
| 10 | allergic | 197 | F | 51.6 |
| 11 | allergic | 41 | F | 100.0 |
| 12 | tolerant ^b)^ | 23 | M | 2.5 |
| 13 | tolerant | 52 | M | 0.3 |
| 14 | tolerant | 31 | M | 0.3 |
| 15 | tolerant | 103 | M | 0.1 |
| 16 | tolerant | 51 | M | 0.2 |
| 17 | tolerant | 30 | M | 0.0 |
| 18 | tolerant | 51 | F | 1.3 |
| 19 | tolerant | 70 | F | 0.0 |

a) Patients with clinical history of CMA, positive skin prick test responses, and *in vitro*–specific IgE to cow’s milk and BLG (Patient 3 - 5) and positive reaction in a milk challenge (Patient 6 - 11).

b) Patients with a clinical history of CMA, but without reacting in a milk challenge.

*) means ± SD of 2 instead of 3 repetitions

**Quantification of serum antibody levels by ELISA**

ELISA plates were coated with 3 μg/ml of BLG (#L01030, Sigma-Aldrich) in coating buffer, pH 9.6 (#421701, Biolegend) overnight, washed with 0.05 % TBS-T once and blocked for 2 h at RT with 0.05 % TBS-T. After washing, sera (diluted 1:2 for IgE, up to 1:100 for IgG4) were added, for calibration, a dilution series of D1 IgE (250 – 3 ng/ml) or D1 IgG4 (4000 – 62.5 ng/ml) antibodies, and incubated for one hour at RT. Detection, development and readout were carried out as described in the ELISA experiments above.

**Inhibition ELISA**

ELISA plates were coated with 3 μg/ml of BLG (#L01030, Sigma-Aldrich) in coating buffer, pH 9.6 (#421701, Biolegend) overnight, washed with 0.05 % TBS-T once and blocked for 2 h at RT with 0.05 % TBS-T. D1 IgG4 was added in increasing concentrations (0.1 nM to 1000 nM) and incubated for one hour at RT. Next, D1 IgE was incubated in concentrations (0.015 nM to 1 nM), for one hour at RT. Bound IgE was detected using HRP-labelled anti-human-IgE antibody diluted 1:6,000 (#A18793, Invitrogen), as described above.

**Basophil activation tests and BAT inhibition**

We followed the protocol previously described.^S14^ Briefly, ficoll-isolated (Ficoll-Paque PLUS, GE Healthcare) PBMCS of healthy donors were IgE-stripped by incubation in lactic acid buffer (13.4 mM lactic acid #W261114-1KG-K, Sigma-Aldrich), 4.09 g of NaCl, 0.186 g KCl in 500 ml of dH_2_O and adjust pH to 3.9) for 4 min on ice. After two washing steps, cells were sensitized with 5 nM D1 IgE in the presence of 10 ng/ml IL-3 (Miltenyi Biotec, Bergisch Gladbach, Germany), for 2 hours at 37 °C. Sensitised cells were seeded into a 48-well plate at a concentration of 1x10^6^ cells per well. Cells were then stimulated with 1000 ng/ml BLG (#L7880, Sigma-Aldrich), control allergen casein 1000 ng/ml (#C7078, Sigma-Aldrich) or medium, in the presence of 2 ng/ml IL-3, for 40 min at 37 °C. In the BAT inhibition experiments, D1 IgG4 antibody (final concentration: 10-100 nM) was added with the BLG. Cells were transferred to FACS tubes and washed with PBS. After centrifugation (300 x g for 5 min), cells were re-suspended in 1 ml PBS and stained with LIVE/DEAD™ fixable near-IR viability dye (#L10119, Invitrogen) for 25 min on ice in the dark. After another washing step, cells were stained with PerCP-Cy5.5-labelled anti-HLA-DR (#560652, BD Biosciences, San Jose, CA, USA), PE-Cy7-labelled anti-FcεRI (#25-5899-42, Invitrogen), BV510-labelled anti-CD123 (#563072, BD Biosciences), and FITC-labelled anti-CD63 (#557288, BD Biosciences) antibodies, and incubated in PBS-BSA for 25 min on ice in the dark. After the last washing step, cells were re-suspended in 175 µl of PBS and measured in MACSQuant Analyzer 10 flow cytometer (Miltenyi Biotec) and analysed with FlowJo software (version 10.0.7, Tree Star, Ashland, OR, USA).

**Flow cytometry and intracellular cytokine determinations**

Cells were IgE-stripped and sensitised with D1 IgE as described above and 1x10^6^ cells were seeded directly into FACS tubes. BLG, casein or medium, as well as 2 ng/ml IL-3 were added and incubated for 6 h at 37 °C with GolgiStop (0.66 µl/ml, #554724, BD Biosciences) and GolgiPlug (1 µl/ml, #555029, BD Biosciences). The tubes were then incubated at 4 °C overnight. Cells were stained with PE-Cy7-labelled anti-FcεRI (#25-5899-42, Invitrogen), BV510-labelled anti-CD123 (#563072, BD Biosciences) and FITC-labelled anti-CD63 antibodies (#557288, BD Biosciences), for 25 min on ice in the dark. After washing with PBS-BSA, cells were re-suspended in 250 µl fixation/permeabilisation solution (#554722, BD Biosciences), and incubated for 20 min on ice in the dark. After washing with permeability wash buffer (#51-2091KZ, BD), cells were stained with BV421-labelled anti-IL-4 (#500826, Biolegend), PerCP-Cy5.5-labelled anti-IL-13 antibodies (#501911, Biolegend) and 50 µl of Brilliant Buffer (#563794, BD Biosciences) for 25 min on ice in the dark. After the last washing step, cells were re-suspended and analysed as described above.

**Statistics**

All statistical analyses were performed using Graph Pad Prism 8 (GraphPad Software LLC). Blood serum data were analysed with Shapiro-Wilk tests to analyse data for normal distribution, Mann-Whitney-U test and spearman correlation. Repeated measures ANOVA with Greenhouse-Geisser correction and Sidak multiple comparison test were calculated for the basophil data. For the cytokine analyses of the basophils, a two-way ANOVA with Greenhouse-Geisser correction and Bonferroni multiple comparison test were conducted. Basophil inhibition experiments were analysed with Friedmann-Test and Dunn’s multiple comparison test for three groups.

**Suppl. References**

S01. Saarinen KM, Pelkonen AS, Mäkelä MJ, Savilahti E. Clinical course and prognosis of cow’s milk allergy are dependent on milk-specific IgE status. J Allergy Clin Immunol. 2005;116(4):869-875. doi:10.1016/j.jaci.2005.06.018

S02. Pecora V, Valluzzi RL, Mennini M, Fierro V, Dahdah L. Debates in Allergy Medicine: Does oral immunotherapy shorten the duration of milk and egg allergy? the pro argument. World Allergy Organ J. 2018;11(1):11. doi:10.1186/s40413-018-0191-6

S03. Keet CA, Frischmeyer-Guerrerio PA, Wessel F, et al. The safety and efficacy of sublingual and oral immunotherapy for milk allergy. *Rev Port Imunoalergologia*. 2012;20(1):66-67. doi:10.1016/j.jaci.2011.10.023

S04. Pajno GB, Fernandez-Rivas M, Arasi S, et al. EAACI Guidelines on allergen immunotherapy: IgE-mediated food allergy. Allergy Eur J Allergy Clin Immunol. 2018;73(4):799-815. doi:10.1111/all.13319

S05. Sato S, Yanagida N, Ogura K, et al. Immunotherapy in food allergy: Towards new strategies. Asian Pacific J Allergy Immunol. 2014;32(3):195-202. doi:10.12932/AP0395.32.3.2014

S06. Niemi M, Jylhä S, Laukkanen ML, et al. Molecular Interactions between a Recombinant IgE Antibody and the β-Lactoglobulin Allergen. *Structure*. 2007;15(11):1413-1421. doi:10.1016/j.str.2007.09.012

S07. Köhler VK, Crescioli S, Fazekas-Singer J, et al. Filling the Antibody Pipeline in Allergy: PIPE Cloning of IgE, IgG1 and IgG4 against the Major Birch Pollen Allergen Bet v 1. Int J Mol Sci. 2020;21(16). doi:10.3390/ijms21165693

S08. Orengo JM, Radin AR, Kamat V, et al. Treating cat allergy with monoclonal IgG antibodies that bind allergen and prevent IgE engagement. Nat Commun. 2018;9(1). doi:10.1038/s41467-018-03636-8

S09. Verhoeckx KCM, Vissers YM, Baumert JL, et al. Food processing and allergenicity. *Food Chem Toxicol*. 2015;80:223-240. doi:10.1016/j.fct.2015.03.005

S10. Upton J, Nowak-Wegrzyn A. The Impact of Baked Egg and Baked Milk Diets on IgE- and Non-IgE-Mediated Allergy. *Clin Rev Allergy Immunol*. 2018;55(2):118-138. doi:10.1007/s12016-018-8669-0

S11. Bu G, Luo Y, Zheng Z, Zheng H. Effect of heat treatment on the antigenicity of bovine α-lactalbumin and β-lactoglobulin in whey protein isolate. *Food Agric Immunol*. 2009;20(3):195-206. doi:10.1080/09540100903026116

S12. Hufnagl K, Afify SM, Braun N, et al. Retinoic acid-loading of the major birch pollen allergen Bet v 1 may improve specific allergen immunotherapy: In silico, in vitro and in vivo data in BALB/c mice. Allergy Eur J Allergy Clin Immunol. 2020:1-5. doi:10.1111/all.14259

S13. Guhsl EE, Hofstetter G, Hemmer W, et al. Vig r 6, the cytokinin-specific binding protein from mung bean (Vigna radiata) sprouts, cross-reacts with Bet v 1-related allergens and binds IgE from birch pollen allergic patients’ sera. Mol Nutr Food Res. 2014;58(3):625-634. doi:10.1002/mnfr.201300153

S14. Zenarruzabeitia O, Vitallé J, Terrén I, et al. CD300c costimulates IgE-mediated basophil activation, and its expression is increased in patients with cow’s milk allergy. J Allergy Clin Immunol. 2019;143(2):700-711.e5. doi:10.1016/j.jaci.2018.05.02

**Supplementary Figures and Legends**

**Figure S1:** **Species-specificity of the anti-BLG D1 IgE and D1 IgG4 antibodies.** For the in-house ELISA 0.1 μg per well of commercial anti-BLG antibodies (#Ab112893, Abcam) were coated onto ELISA plates and incubated with the following samples: raw cow milk, ultra-high temperature processed (UHT) cow milk, sheep milk, or goat milk (x-axis). For detection of BLG, D1 IgE (1 μg/ml) or D1 IgG4 (0.25 μg/ml) antibodies were added and detected with HRP-labelled anti-human IgE and gG4. For quantification (y-axis), BLG was serially diluted (0.05 – 2.5 μg/ml) and values compared hereto. The commercial sandwich ELISA set for BLG was used according to the manufacturer´s instruction and quantified using provided standards. Columns represent the mean of duplicates from three experiments, error bars indicate +/- SD between experiments.

**Figure S2:** **PIPE-cloned D1 IgE and D1 IgG4 for quantification of natural antibodies to BLG in patients’ sera.**

BLG was coated onto ELISA plates and incubated with sera (diluted 1:2 for IgE and between 1:5 and 1:100 for IgG4 depending on sera IgG4 content) of CM-sensitized patients (allergic, n=9, or tolerant in milk challenge, n=8). Natural bound IgE (A) and IgG4 (B) was quantified comparing to dilution series of D1 IgE or D1 IgG4, all detected by HRP-labelled anti-human IgE or anti-human IgG4. Data points represent the mean of three independent experiments. Differences were calculated by Mann-Whitney test: U = 11, p = 0.0089); *P < .05, **P < .01, and ***P < .001.

**Figure S3:** **Basophil gating strategy in flow cytometry.** Peripheral blood mononuclear cells (PBMCs) of one exemplary donor are shown, stained with anti-HLA-DR (PerCP-Cy5.5), anti-FcεRI (PE-Cy7), anti-CD123 (BV510), anti-CD63 antibodies (FITC). Basophils were gated as CD123^+^FcεRI^+^HLA-DR^-^, after excluding doublets and dead cells.
